# Examining the Impact of Pyramid Model Implementation to Deepen Social and Emotional Supports for Enrolled Children Within Early Intervention

**DOI:** 10.3390/healthcare13050515

**Published:** 2025-02-27

**Authors:** Margo Candelaria, Kate Sweeney

**Affiliations:** Parent, Infant, Early Childhood (PIEC) Program, Innovations Institute, University of Connecticut School of Social Work, 38 Prospect St, Hartford, CT 06103, USA; kate.sweeney@uconn.edu

**Keywords:** Pyramid Model, Part C Early Intervention, Implementation Science, Social and Emotional Development, Individuals with Disability Education Act (IDEA)

## Abstract

**Background/Objectives:** Early social and emotional well-being is crucial for child development with life-long outcomes. The Individuals with Disabilities Education Act (IDEA) Part C Early Intervention system, which federally mandates special education services for children ages birth to three, is one place where identifying and addressing early social emotional needs could be strengthened. Focusing on an implementation science approach, the Pyramid Model (PM) is examined to see how the model’s targeted implementation efforts over many years enhance a system’s capacity to identify and address social and emotional well-being in one state over five years. **Methods**: Implementation science methods were used to evaluate the impact of PM implementation over time in four independently operating sites within one state, emphasizing the use of coaching to support practice change, as well as fidelity tools and examination of system changes over time. **Results**: All sites increased capacity for PM implementation. Results varied by site but all sites were able to demonstrate improvement and higher fidelity implementation by the last year. **Conclusions**: The PM is an effective model to use within IDEA Part C programs to enhance capacity to identify and address social and emotional needs of children and families receiving services. Implementation factors are needed for success, including leadership support, organizational dynamics (e.g., creating dedicated time for staff to engage in training, external and internal coaching, and case reviews), committed funding, and dedicated support for implementation and evaluation.

## 1. Introduction

The earliest years of life present a unique opportunity to lay the foundation for healthy development. It is widely considered a time of great growth and vulnerability. Decades of research on early childhood has underscored the impact of the first five years of life on future social and emotional development [1] and lifelong health outcomes [2,3]. Improving meaningful long-term outcomes for children depends on several intentional additions to typical service provision, including (1) promoting supportive relationships for young children, (2) intentional teaching of social and emotional skills and strategies, and (3) and reducing sources of stress for children and families [4]. Early experiences can either support or impair all domains of development, and these early developmental opportunities establish a critical foundation for children’s academic success, health, and general well-being with a safe, stable environments identified as protective, and toxic stress identified as detrimental to early childhood development [5,6]. Nurturing caregiver–child relationships provide protection and mitigating deleterious effects of early exposure to trauma and adverse events [4,7,8,9].

Policy makers and early childhood experts in a range of states and localities are partnering with infant mental health experts to support a focus on implementing and evaluating a range of interventions designed to improve school readiness and developmental outcomes for the most vulnerable of our population: young children living within the context of developmental delay. Efforts are also underway to improve the capacity of early intervention and education programs to promote optimal development for participating infants and toddlers through a variety of quality improvement initiatives. The American Academy of Pediatrics has emphasized the promotion of early relational health and positive and nurturing relationships to address the impact of toxic stress [10]. In addition, there is ample evidence that fostering positive relationships between young children and their caregivers is associated with improved developmental outcomes such as talking [11,12], reading [13,14], and playing [15,16]. 

There is growing recognition that children who are especially vulnerable benefit greatly from additional interventions and support, especially those that promote healthy social and emotional development and support positive interactions in supportive services and programs throughout their academic experiences [17]. There is evidence that children exposed to social emotional interventions have increased social and emotional competence and self-regulation compared to those who do not receive interventions [18]. Between 9.5 and 14.2% of children between birth and five years old experience social and emotional problems that negatively impact their functioning, development, and school-readiness [19]. Based on the 2016 National Survey of Children’s Health, approximately 20% of children ages 3–5 are not considered ‘On Track’ for the Social Emotional Development and Self-Regulation domain of Healthy and Ready to Learn [20]. Despite research that supports identification, early intervention, and treatment, many young children do not receive screening, services, or supports. Inadequate screening prevents recognition of social, emotional, and behavioral problems. Less than one percent of young children with emotional behavioral problems are identified [21]. Therefore, programs that initiate efforts to intentionally address promotion of social and emotional well-being in very young children are warranted. One key federal program where social and emotional concerns should be supported within service delivery is the IDEA Part C Early Intervention program.

### 1.1. IDEA Part C Early Intervention

The Individuals with Disability Education Act (IDEA) legislates special education services for children 0–21 in every state. Part C of IDEA, also known as Early Intervention (EI) services or the Infants and Toddlers Program (ITP), is the part of the federally legislated special education services dedicated to children from birth to age 3. IDEA Part C services are required to be offered within each state. States have purview to create individual eligibility requirements. Typically, children need to demonstrate development delays and/or significant risk for developmental delays to qualify for services. There are five developmental areas assessed when determining eligibility: cognitive skills, motor skills (gross and fine), language skills (receptive and expressive), self-help skills, and social emotional skills. In order to comprehensively cover all domains of development, Part C teams are made up of multi-disciplinary providers (e.g., special educators, speech and language therapists, occupational therapists, physical therapists, nurses or health providers, and mental health providers) who often work collaboratively and provide cross-disciplinary services. After eligibility is determined, services are documented in an Individualized Family Service Plan (IFSP) where the child’s developmental levels and goals for intervention are documented, along with the families’ priorities, and any other referrals or linked services. Services should be offered in the natural environment, often in a child’s home or child care location.

An emerging trend has state Part C teams moving from a medical service model (e.g., focused on the individual patient) to a family coaching model, whereby the preponderance of intervention focuses on providers coaching parents and primary caregivers to provide intervention to the child. For example, a physical therapist would coach a caregiver to provide needed exercises and activities to the child and the caregiver would practice these in between visits with the provider. Recent meta-analysis [22] has shown the impact of this approach, demonstrating that parents and primary care givers of children receiving services learned to use strategies and skills at “significantly higher rates” when a coaching approach was used compared to services when it was not. Similarly, children made more progress, and at a quicker rate.

The Office of Special Education Programs (OSEP) has initiated State Systemic Improvement Plans (SSIPs) for state special education programs that serve as guidance for programs to systematically implement evidence-based practices (EBPs). State Part C teams have SSIP plans separate from other parts of special education services. SSIPs are multi-year plans, with many states now in their third round of multi-year SSIP implementation cycles [23]. Of the 56 states and territories reporting on Part C SSIP activities, 29 (52%) have elected to include address Infant and Early Childhood Mental Health (IECMH) within their SSIP plan, and 11 (20%) states specifically identified the Pyramid Model (PM) as their chosen EBP [24].

It should be noted that the Part B section 619 of IDEA federally legislates special education services to children ages 3 to 5 with services offered through an Individualized Education Plan (IEP). Accordingly, the IEP is a legal document that follows federal guidelines for the provision of special education services through public education programs for students for ages 3 through 21. Part B section 619 specifically refers to children ages 3–5 and their IEP services. Some states and localities provide early intervention services for Part C as a separate program and some states combine Part C and Part B 619 services into a Birth–5 program. The term “early intervention” is often used interchangeably in Part C and the Part B 619 literature and data are often reported across programs.

### 1.2. Part C and Addressing Social Emotional Needs

Although evaluation of social emotional skills is required in Part C, it is often neglected due to lack of training and minimal understanding of infant and early childhood mental health [25]. Analyzing data over 10 years, the study found that social and emotional challenges were cited as the formal reason for referral 4% of the time. However, 32% of parents reported having difficulty managing their child’s behavior. About 32% of children were described by parents as highly distractible, 13% as not persisting in tasks, 10% as having very challenging behavior, 11% as aggressive with other children, and 5% as very withdrawn. This suggests that within Part C EI programming, more attention is needed to infant and early childhood mental health. The U.S. Department of Education Office of Special Education Programs measures and reports data from Part B 619 and Part C on three child outcomes: social relationships, including getting along with other children and relating well with adults; use of knowledge and skill, referring to early literacy and math skills; and taking action to meet need, such as feeding, dressing, and self-care. Only 53% of children exiting Part C Preschool in 2020–2021 school year and 56% of children exiting Part B Preschool exited at or above age expectations for building and maintaining social relationships with adults and their peers [24]. This is part of a national trend where the percentage of children exiting Part B and Part C Preschool programs at or above age expectations has declined in all three outcomes in the last 5 years. The proportion of children 0-2 with disabilities who meet age expectations for social relationships has declined from 58% in 2016 to 53% in 2020. 

A recent survey of Part B 619 and Part C programs by the National Center for Children in Poverty demonstrated that although 31 states have identified social emotional concerns as the focus of the SSIP work, there are still significant gaps in addressing social emotional concerns in early intervention [26]. Thirty states reported recommending conducting social emotional screening and recommending screening tools, but only eight states require using a specific social emotional screener. If children are not routinely screened, the ability to accurately identify children with social emotional needs is diminished. The same survey also reported that the majority of states do not have specific expertise in infant mental health on evaluation teams. In addition, states had varying capacity to provide or connect parents with services within the EI programs and most often referred out.

For young children from stressed families and communities, early intervention and education programs offer an opportunity for safety and nurturance. They also provide opportunities for children to develop social and emotional skills from a calm and predictable environment and for parents to receive support and linkage to critical resources. Research shows that children who are fortunate to experience high-quality care and early intervention enter school well prepared and are more successful throughout school and later in their lives [27]. 

### 1.3. The Pyramid Model (PM) for Promoting Social and Emotional Competence in Infants and Young Children

The PM is an evidence-driven framework that promotes the social and emotional development and school readiness of young children from birth through age 8 and has been found to help teachers improve social/emotional and behavioral outcomes for children [28]. There is a significant evidence base that grounds the structure and strategies of the PM. The PM is a multi-tiered system of support that promotes the healthy social and emotional development of children from birth to age eight across diverse early childhood settings, including infant and toddler and preschool classrooms, family childcare, Head Start and Early Head Start, and early intervention. The framework was developed to support early childhood educators to promote social emotional development, prevent social emotional delays and behavior problems, and provide individualized intensive interventions for children who need behavioral support [29]. Key practices within each tier of the PM includes building positive relationships with children and families and creating nurturing and supportive environments (Tier 1), individualized teaching of social emotional skills that may be lacking (Tier 2), and the use of functional behavior assessment to determine the meaning of behavior and develop a plan that meets the specific needs of the child (Tier 3) [29]. Several researchers identified that an increase in PM fidelity scores, which is indicative of an increase in use of PM practices, was associated with a decrease in challenging behavior [28,30,31,32]. In addition, children who were in the treatment condition where their teachers received training and support for PM practices also often demonstrated increased social skills [28,32,33]. 

Regarding Part C EI specifically, the PM has created specific content and adaptations for working within the EI system [34]. Application of the PM focuses on supporting early intervention practitioners to:

(1) Build collaborative partnerships with families;
(2) Use family coaching strategies;
(3) Provide families with skills and knowledge in responsive caregiving and nurturing relationship;
(4) Build family competence in promoting social emotional development;
(5) Provide families tools and strategies to address challenging behavior.

Although there are a number of Part C specific materials and guidance documents offered through NCPMI [35], and state reports that include examples of Part C implementation and outcomes [36], there are to date no research studies examining PM implementation in Part C programs specifically. This paper presents a contribution to the field by sharing the evaluation of PM Part C implementation, focused on an implementation science perspective.

### 1.4. PM and Implementation Science

PM implementation is rooted in implementation science [37,38]. Implementation science is a framework that guides systems changes [37,39] and focuses on supporting systems through four stages of implementation—exploration, installation, initial implementation, and full implementation. Implementation science also includes examination of implementation drivers, which are the engine of change [37,39]. As with the implementation stages, drivers are fluid and interact with one another to lead to consistent implementation and positive outcomes. Implementation drivers have been categorized as competency, organization, and leadership supports. Competency supports include training, coaching, and staff selection. For the PM, training and coaching address competency. Each individual program and locality brings its own organizational and leadership structure and climate. Organizational support includes administrators who can change practice, systems interventions that are in place to help providers make change, and decision support data systems. State leadership support is a strong driver for state Part C PM implementation work. Furthermore, the implementation science model has specific applications within early childhood systems and specifically is considered foundational for the PM [23,40].

### 1.5. Purpose

The intent of this paper is to provide an example of how PM implementation was infused into four jurisdictions as part of a state’s Part C SSIP plan from 2017–2023, focusing on data and outcomes. This work included an evaluation of the implementation process and outcomes, focusing on the implementation science approach. Successes and challenges will be highlighted throughout, including the need to adapt to local infrastructure and culture. This process was initiated before ITP specific content and materials were created by the National Center for Pyramid Model Innovations [35] (NCPMI), the federally funded PM technical assistance center. Thus, this demonstrates the evolution of the local PM implementation process in ITP over time as the PM national work also evolved to create ITP specific approaches. This paper will focus on the evaluation process and outcomes. For greater details of the implementation of programmatic processes over time, see [41]. Overall, it was expected that increased PM implementation would take place over time as demonstrated by engagement in training and coaching, examination of fidelity tools over time, and changes in program practices such as routine use of PM practices such as screening for social and emotional needs and ongoing implementation of an embedded local PM leadership team for Part C teams.

## 2. Materials and Methods

### 2.1. State Context

The PM model was implemented as part of the state SSIP plan in a mid-Atlantic state. Four jurisdictions were selected that reflect diverse areas of the state including: location A—a small, predominantly white, rural jurisdiction in the northern part of the state; location B—a mid-sized semi-rural, predominantly white, jurisdiction in the western central part of the state; location C—a suburban, racially diverse jurisdiction in the central part of the state; and location D—the largest and most racially diverse jurisdiction in the central part of the state that ranges from urban to rural. See Table 1 for details. These jurisdictions were selected through a combination state leadership selection based on identified needs and capacity and jurisdictional leadership agreement to participate. It should be noted that the sites were introduced to the PM in 2015, with them engaging in exploration of the model and initial training of content in the time from 2015–2017. The authors came to the work in 2017 and this study reports on activities from 2017 through 2023.

### 2.2. State SSIP Plan

**Figure 1:**
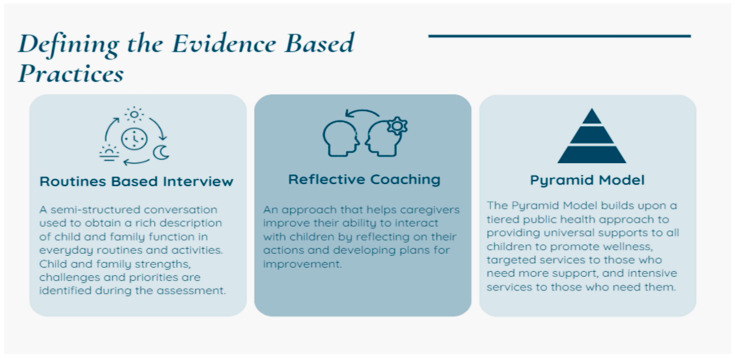
SSIP plan social emotional EBPs.

**Figure 2:**
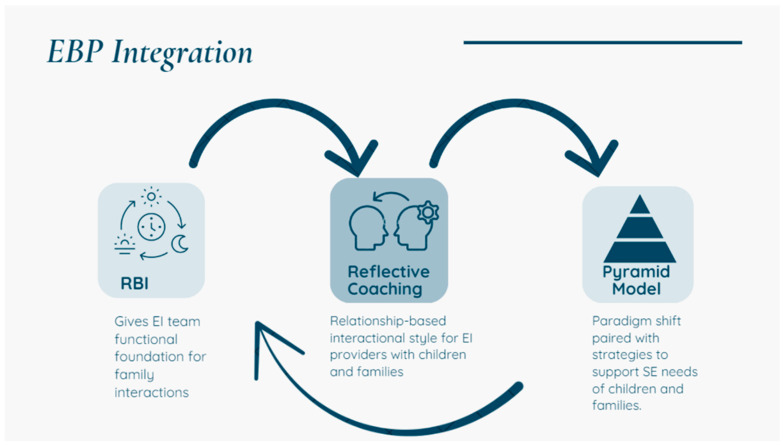
SSIP EBP integration.

Within this effort, the state’s Department of Education led and funded a multi-year initiative to engage state leads for three targeted evidence-based practices: PM, Routines Based Interview (RBI; [47]), and Reflective Coaching [48,49]. Applying the structure and processes of implementation science, the state leads for the three EBPs worked collaboratively to support the local Part C teams to receive training, coaching, and internal policy planning for implementation and sustainability of the three EBPs. The intended outcome was integration of supports for IFSP engaged children, their families, and their child care providers with respect to social and emotional needs and concerns. As reflective coaching has spread across Part C teams nationwide, Part C provider family conversations have naturally shifted. Through holding a focus on families’ concerns (joint planning) and embedding strategies within the family’s daily routines (observation, action), concerns or questions about challenging behavior are more organically expressed and shared. This provides a natural opening for families to seek guidance from their providers around social and emotional strategies. Since Part C EI services are, by design, delivered by a mixed discipline workforce, providers report a varying range of comfort and confidence in addressing social emotional content that comes up within sessions, such as behavioral strategies, mental health, family trauma, and caregiver stress. The PM implementation operated within this overall framework and took place in conjunction with RBI and Reflective coaching. Figure 1 and Figure 2 below demonstrate the framework and process that guided the work.

### 2.3. University Partnership

The PM implementation was facilitated at all sites by external partners from a local university. University partners had both general implementation and evaluation expertise, as well as specific experience and knowledge of PM implementation and evaluation practices within a range of early care and education settings. The university partnership team worked closely with NCPMI and National Pyramid Model Consortium (PMC) partners to ensure alignment with best practices and current and evolving materials. This model of having implementation guidance is supported by implementation science practices [50] and was consistent with the core principles of implementation support approaches including co-creation, ongoing improvement, and sustaining change [51,52]. The university partnership was consistent throughout the implementation process with the implementation and evaluation leads staying the same throughout. This consistency allowed for trust to build on state and local site teams. Additionally, university partners developed deep understandings of local structures, needs, and populations served, as well as the implementation of historical knowledge among the various participating teams and state leads.

### 2.4. Measures

The measures used focused on demonstration of fidelity in implementation and tracking of activities. For this paper we focused on training and coaching activities, implementation fidelity at the local level, and changes in practices.

#### 2.4.1. Training Activities

Training and coaching activities were collected via entry into a statewide PM data collection system (The creation of this state system pre-dates the Pyramid Model Data Implementation System). Training information included content and location of trainings that state approved trainers entered. The Impact of Training and Technical Assistance [53] (IOTTA) was used pre/post training to evaluate each training assessing for trainee satisfaction, learning, and intent to use content in the future. The IOTTA was distributed and collected at the close of both in person and virtual trainings via web-based links.

#### 2.4.2. Training Approach

Training efforts focused initially around introducing the individual EBP models to jurisdiction leadership teams. As noted in the above chart, due to the range in size and other variables for each of the sites, we worked closely with each leadership team to identify individualized training plans that would meet the needs of their jurisdictions. Some jurisdictions first initiated PM implementation with local pilot sites or cohorts, whereas others opted to have all staff initially trained in the model. As often happens with PM training for providers and systems, the initial training opens up a paradigm shift for many. It asks that all professionals within their various roles in early childhood and family serving systems see the value in embedding supports for social and emotional assessment and needs of children in their care. Accordingly, throughout this multi-year effort, many site teams requested training content to go deeper based on trends of need they were seeing within their cases, (e.g., caregiver mental health, anxiety and trauma). Additionally, beyond the training that all staff received within engaged teams, we worked specifically with identified internal coaches to support them via a series of trainings and coaching sessions to support colleagues with both the Pyramid Model framework, as well as specific guidance for embedding this approach into IFSP service delivery. Additional training topics beyond the core Part C PM content included:

i Equity in Early Childhood
ii Pyramid Model Within Part C—An Introduction
iii Understanding Early Childhood Anxiety Through the Pyramid Model
iv Understanding Early Childhood Trauma Through the Pyramid Model
v Social and Emotional Assessments and Screeners
vi Integrated approaches to coaching colleagues (this was done in partnership with the other EBP leads for RBI and Reflective Coaching) Part C PM Sustainability.

#### 2.4.3. Coaching

In the years 2017–2019, during initial implementation, university partners emphasized how to engage in coaching. The Coaching Practices Rating Tool [48] (CPRT) was used annually to assess coaching capacity. The CPRT was created as part of the Reflective Practice created by Rush and Sheldon [48], and was used across EBPs to get a general sense of uptake of coaching skills and practices among the program staff. At the time that EBP implementation was started, there was not a Part C specific coaching measure for PM. Thus, the CPRT was used by the state leadership to examine coaching capacity within programs as it applied to all EBPs.

Coaching activities of the university partners who provided external coaching to identified internal coaches within jurisdictions were also tracked in the state data system. Coaching information was based on coaching logs published by NCPMI that state approved coaches entered into the statewide data system after coaching sessions. Coaching frequency, content, and methods were tracked for each session.

#### 2.4.4. Implementation Fidelity

The Benchmarks of Quality (BoQs) is the primary fidelity tool of the PM [54,55]. The BoQs include six critical element domains (Leadership Team, Staff Readiness and Buy-In, Family Engagement, Building Staff Capacity, Providing Interventions to Children with Persistent Challenging Behavior, and Monitoring Implementation and Outcomes). Together the critical elements consist of a total of 30 indicators. BoQs were collected by local leadership teams. It should be noted that the Part C-specific BoQs provided by the NCPMI evolved over time. When this implementation work started there was a BoQ with 24 items, which was used in 2017. NCPMI then released an updated Part C BoQ in 2018. Thus, while the BoQs were used over time, the same version was not used at all time points. However, the content is generally consistent. In addition, the measurement approach, examining the % of items in place and targeting coaching to items not in place, was consistent over time.

The Early Intervention Pyramid Practices Fidelity Instrument [55] is now considered the primary fidelity tool for Part C PM implementation. However, the EIPPFI was created after this project was started and was not in use in the state until 2021. Training on how to use the EIPPFI was initiated in 2021 but was not fully tracked as implementation of the tool was gradual and required a shift in approach and another layer of data collection that some sites found burdensome.

#### 2.4.5. Practice Changes

An additional indicator of PM implementation is systems change over time. Over time we focused on two components of systems change that would have a long-term impact. The first targeted system changed was having an ongoing functioning leadership team that met independent of other teams and oversaw PM implementation. The second was implementation of social emotional screening. Sites were encouraged and trained on implementation of social emotional screening to identify social emotional needs. Engagement of targeted versus universal screening was a local decision based on capacity, but universal screening was strongly encouraged. Although all Part C evaluation teams are required to do a cursory social emotional screen, the intention was to use a more in-depth and specific social emotional screener that was dedicated to identifying social emotional concerns, compared to having a few questions within a global developmental screener. This would allow for better identification of concerns and allow for more specific creation of goals for intervention.

#### 2.4.6. Stages of Implementation

From an implementation science perspective [37,38], implementation progress can be monitored over time as a way to assess progression through stages. Although there are several models of implementation stages, this project utilized the implementation stages model which moves progressively from exploration, installation, initial implementation, and full implementation [38,40]. This is the model affiliated with the PM [56]. Progress can be slow and staying in one stage is not necessarily indicative of lack of progress. In addition, the need to revisit and re-do work is sometimes necessary due to staff changes, leadership changes, and policy changes over time. All four SSIP jurisdictions were in or beyond the exploration stage at the end of 2017. As noted above, sites had from 2015 to 2017 to engaged in exploration of the model, committed to implementation and engaged in initial training activities. Thus, tracking of implementation in 2017 began with installation.

## 3. Results

### 3.1. Training

#### 3.1.1. Training Activities

Training took place for most sites each year. As demonstrated below in Table 2, sites varied in trainings over time, reflecting local interest and needs. For example, site A asked for a training focusing on trauma and infants born addicted to substances because they were seeing this in greater numbers in their caseloads. Screening for trauma was a repeated request. In addition, applying PM practices to the Part B 619 programs and application in a Birth–5 system was a common request. This demonstrated expansion of PM activities beyond Part C and integration with other systems.

#### 3.1.2. Training Evaluation

All trainings were rated after each session using the IOTTA. Examination of all IOTTAs across all years indicated strong evaluation scores with training individuals, training methods, and training content rated highly. In addition, for each training, increases in knowledge were demonstrated, and intent to use content in their work was endorsed.

### 3.2. Coaching

#### 3.2.1. Coaching Practices

Examination of the Coaching Practices Rating Scale (CPRS) was examined for 2017, 2018, and 2019. Examination of scores indicated positive changes over time. In 2017, no scores were above three, compared to 2019 where ten of the fourteen scores were above 4 and the remaining four were above 3.86. See Table 3 for all scores over time.

#### 3.2.2. Coaching Content

Coaching Content focused on implementation practices and used the BoQs as a guidance tool. Integration of social emotional screening was increased over time. In addition, the EIPPFI was introduced to some teams as a tool in 2021 and EIPPFI implementation was incorporated into coaching. As demonstrated in Table 4 below, the frequency and content of the external coaching by the university partners varied by site. Site C engaged in minimal external coaching per their request as they were focused on implementation of other EBPs. Per the reports, they maintained internal coaching and continued use of BoQs.

#### 3.2.3. Coaching Fidelity

**Figure 3:**
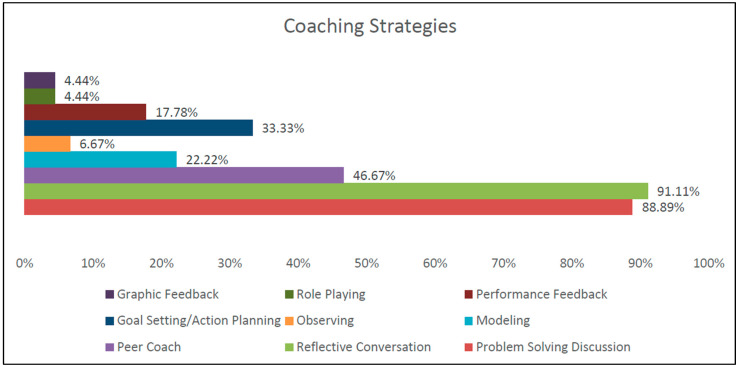
PM external coaching strategies.

**Figure 4:**
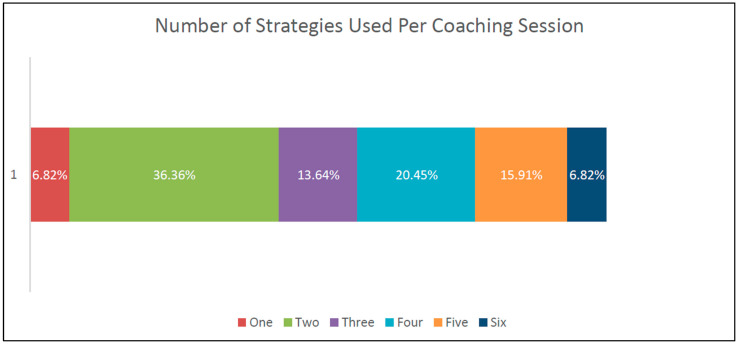
PM number of coaching strategies per session.

Coaching fidelity of the external coaching provided by the university partners was examined by tracking coaching strategies used and identifying number of strategies used per session, as documented in coaching logs. As can be seen in Figure 3, most coaching sessions used the strategies of Problem-Solving Discussion and Reflective Conversation (over 88% and 91% respectively). Almost half of coaching sessions also utilized peer coaching. These results are consistent with systems level coaching as coaches are not present in a classroom or a home visit but relying on providers to convey activities. Use of peer coaching is an excellent way to increase coaching capacity for local coaches. Role Playing and Graphic Feedback were the least used. In addition, as can be seen in Figure 4, over 93% of coaching sessions used two or more coaching strategies per session, demonstrating a variety of approaches used during coaching sessions.

### 3.3. PM Implementation Fidelity—Benchmarks of Quality (BoQ)

**Figure 5:**
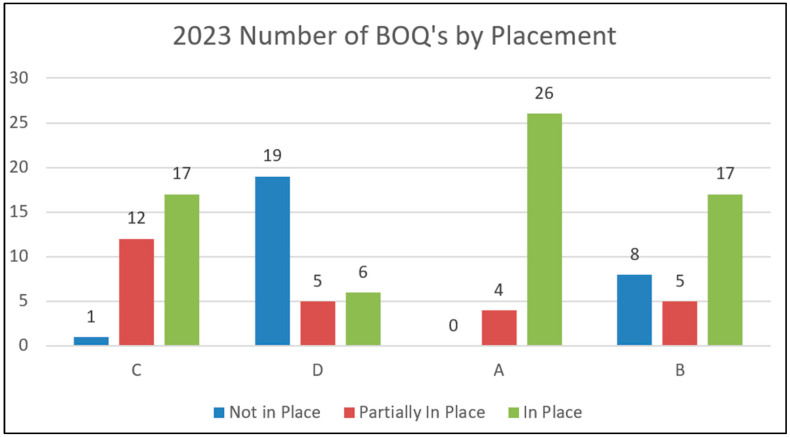
Number of BoQ items in place by site.

**Figure 6:**
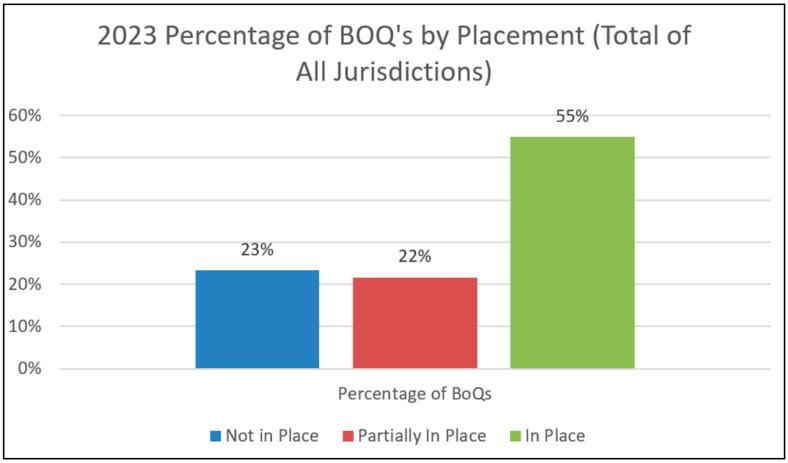
Number of BoQ items in place across sites.

Each SSIP county completed a BoQ in 2023 as their final BoQ for the SSIP cycle. Figure 5 shows the number of BoQ items in place by 2023. In most of the sites, a majority of the implementation components were fully in place. For site A, 26 were in place, and for sites B and C, 17 were in place. However, site D only had six in place. This is in contrast to prior years where site D had far more items in place. This change demonstrates the impact of program-wide policy and procedure changes in response to the BoQ review and action planning, as well as the implications of staff turnover in a significantly larger jurisdiction as a barrier to progress in the initial years of implementation progress. Overall, the results show strong PM implementation as their SSIP time came to a close. Figure 6 also shows that collectively across sites, 55% of items were in place and 22% were partially in place, with only 23% of items not in place.

**Figure 7:**
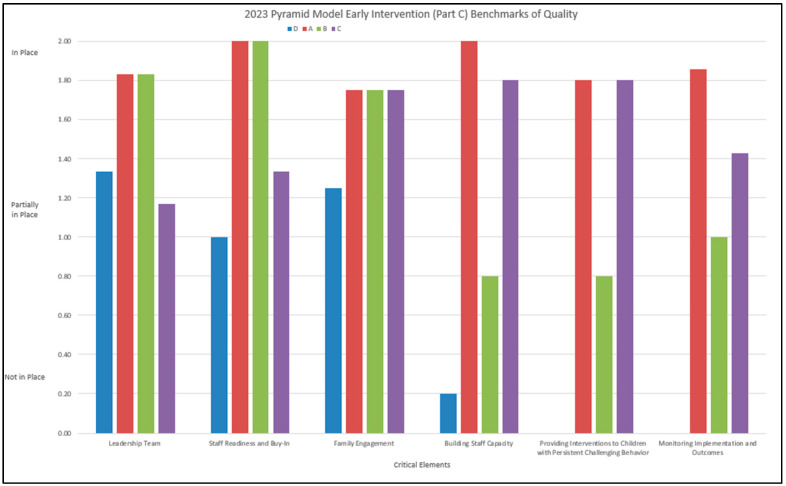
Average score of critical elements by site.

Figure 7 shows BoQ scores by site for each critical element. Average scores by critical element show that site A had an average of 2 (out of a total possible score of 2) in both Leadership Team and Building Staff Capacity. Their average score was at least one in all critical elements. Site B had a 2 for Staff Readiness and Buy-In and just under 2 for Leadership Team and Family Engagement, as well as a 1 for Monitoring Implementation and Outcomes. Site C had a 1.17–1.8 for all Domains. Site D ranged from 1 to 1.33 for Leadership Team, Staff Readiness and Buy-In, and Family Engagement. Overall, Leadership Team, Staff Readiness and Buy-In, and Family Engagement had the highest scores. Every site had an average score of at least one in every critical element, except for Providing Interventions to Children with Persistent Challenging Behavior in Frederick County (*m* = 0.70).

In order to examine the progress of PM implementation in Part C over five years we looked at local site BoQs from 2017 to 2023. BoQs were available for the years 2017, 2019, 2020, 2021, and 2023. They were not collected in 2018 as we were informed of the new Part C BoQ that year and focused on coaching to adapt to the new form, which was first collected in 2019. They were not collected in 2022. For this state, January of 2022 represented the return to schools after over a year of being on lockdown due to the COVID-19 pandemic. The adaptation back to full in person services was an adjustment and teams struggled to focus on data collection. Those results are briefly summarized below in Table 5. Note that in 2017 and 2019, site D reported multiple BoQs across five regions, as they are a large jurisdiction. The numbers below reflect the lowest scores that were predominantly reported in aggregate (e.g., if one region scored 0 and another scored 1, we used the 0). In addition, this was a prior version of the BoQ that only had 24 indicators. Furthermore, although there were two BoQs administered for each county some years, we focused on the last administration for comparison purposes. For site B, there were several indicators missing from the second BoQ. To account for that, we have included the number of items reported (13 indicators), and scores are reflective of these 13 reported items. As can be seen, the ongoing PM implementation work has led to increases in the percentage of in place indicators over time. Despite these differences, the methodological approach of examining the % of items in place is consistent. For all scenarios (13, 24, 30 items), understanding the % of items in place is the goal. Site B only completing 13 items could imply there were challenges implementing those items, but information about the missing data was not explained by the site.

### 3.4. Practice Changes

**Figure 8:**
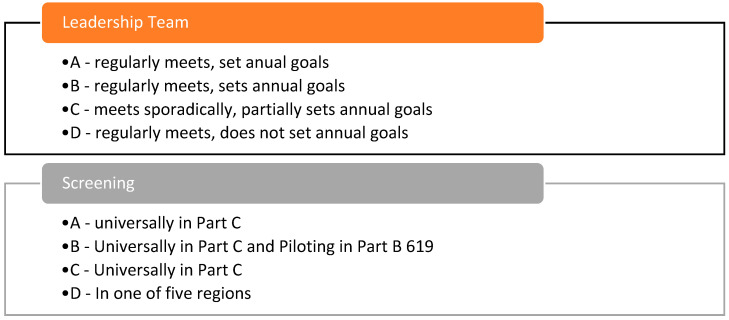
Practice change outcomes.

Focusing on the PM leadership team and screening practices, all sites made progress in making practice changes. In 2023, all sites reported having leadership teams that were still functioning, although site C only reported meeting sporadically rather than regularly. Despite this, site C reported partially setting annual goals whereas site D did not. Regarding screening, all sites were engaging in screening in 2023. Sites A, B, and C reported universal screening which was a primary goal of the PM implementation. In addition, site B was also integrating screening into their part B 619 system as well. Site D reported screening but was only doing so routinely in one of their regional locations. These outcomes are depicted in Figure 8.

### 3.5. Implementation Progress

From an implementation science perspective [37,38], the Pyramid Model implementation in the Part C SSIP jurisdictions for the state advanced over the five years. We examined stages of implementation over time from exploration and installation, through initial implementation to full implementation. Exploration phases were accomplished before this university partner team was engaged. Thus, exploration is not reflected in these results. As can be seen in Table 6, every team moved forward in implementation over time. Although all criteria for full implementation were not yet in place by the end of the five years, two jurisdictions are emerging toward full implementation. This amount of progress is expected as all teams have engaged in ongoing self-analysis, programmatic changes, and making data-driven decisions that inform systems change. As expected, implementation progress varied across sites, with sites A and B experiencing fewer challenges with implementation.

## 4. Discussion

This project sought to examine implementation processes and outcomes to determine if PM implementation would lead to changes within Part C EI programs that enable programs to more successfully address social emotional concerns among young children with disabilities and/or developmental delay. This project was rooted in implementation scienced and focused on implementation drivers, fidelity of implementation, implementation phases, and capacity to create and sustain programmatic changes. Overall, PM implementation within Part C early intervention programs was successful, but variable by site. All sites demonstrated progress in the stages of implementation and increased fidelity scores as measured by the BoQs, the primary program-wide PM fidelity instrument [54,57]. By the end of year five, three of the programs had 57–87% of BoQ items in place, compared to 50% and under for all sites in year one. One site only had 20% in place in year five but had 57% in place in year four, with local changes interfering with year 5 implementation. Leadership Team, Staff Buy-In, and Family Engagement were all strongly in place after several years of implementation, suggesting these foundational items can be maintained over time. These items are what needs to be in place first before deeper implementation can be taken on. Building Staff Capacity, Providing Interventions, and Monitoring Implementation and Outcomes were at least partially in place for three sites but clearly were more challenging to achieve and maintain. This is potentially related to staff turnover and difficulty owning the data processes, with sites preferring to rely on university partners for data monitoring and interpretation. All sites reported having an internal PM leadership team that convened to complete the fidelity tool and make decisions related to implementing action plans for their programs, with three of the four sites continuing to meeting regularly and one meeting sporadically. This indicates sustainability of the leadership team, which is fundamental to maintaining implementation. All sites engaged in some level of internal PM coaching by 2023 and all sites reported maintaining use of the PM. However, only two sites were actively screening for social emotional needs in 2023, suggesting this practice change was harder to maintain over time.

Individual success was highly dependent on local structure and ability of leadership to incorporate PM more fully into program practices. Smaller sites were observed to have more control over making and sustaining changes. Large sites had more challenges and faces for bureaucratic challenges in implementation changes across sites. Site D in particular, which had five local regions within their locality, had to shift to focusing implementation within one region to have more control over implementation, with the intention of spreading implementation to other regions later. This is consistent with the implementation science theory that leadership and organization drivers highly influence implementation success [58].

All sites were supported at the state level and state funding supported implementation efforts. The state leadership team created a structure that supported the sites for implementation of all three EBPs through monthly meetings, collective goal setting, and time to learn from and discuss processes with each other. Furthermore, the state paid for the university partnership work as the PM lead. They also funded other EBP leads for Routines Based Interview and Reflective Coaching, which reduced the burden from local programs. As the SSIP is a multi-year project by design, the state also embraced and supported that implementation is an ongoing process that can take 3–5 years to see change [38,40]. This mindset was crucial for allowing programs to take their time and be thoughtful, as well as not be discouraged by lack of progress. The state leadership team that initiated the SSIP plan was deeply committed to the PM and saw the PM as fundamental to addressing social emotional need within Part C (see Sweeney and Candelaria, 2025 [41] for statements from the state leadership at the time), again demonstrating the importance of leadership as an implementation driver [39].

There were several challenges that impacted successful implementation. One challenge was staff fatigue with multiple local and state requirements. Although the state and local leadership were supportive, other competing demands coming from the state and local systems taxed and overwhelmed staff. As noted above, the state moved to a family coaching model during the early years of implementation. In addition, implementing all three EBPs simultaneously was challenging. Despite efforts to encourage and facilitate integration, sites reported feeling they competed with one another and often focused on one EBP more than the other during some years.

Also, in some jurisdictions, staff models made engaging in PM and other EBP activities challenging. As it is a multi-disciplinary workforce, not all providers worked for the same agency. Also, some agencies required documentation of all time as case-related time, and it could be difficult to justify or get approval for time engaging in coaching. For internal coaches, where the coaching requirements were higher, justifying and getting approval for so much time not engaged in case-related activities was a challenge. This was less of an issue at sites that allowed for internal coaches to have dedicated time with their schedules to support these activities, and/or created administrative positions for these roles.

Staff turnover remains an ongoing issue, and was particularly difficult throughout the COVID 19 pandemic. As demonstrated in the training data, there were annual trainings for new staff in the later years to support the integration of newly hired employees into the program-wide implementation efforts. This slowed implementation progress and those with historical knowledge sometimes moved on. This also led to changes in approach as there were changes in internal coaches over time and leadership changes that could attempt new approaches. Throughout this project, the university partners attempted to support sites and maintain consistency as much as possible.

There were also several limitations to PM implementation over time worth noting here. Although the state funded efforts, funding was limited and without increased across years. As the university partners experienced cost of living salary increases over time, this led to less coverage of time from the university partners. Although internal site independent maintenance increased over time, and external support theoretically decreased over time, the needed time from university partners remained stable. This was partially due to staff turnover, requiring supporting new staff. Additionally, as NCPMI created more Part C specific content and material, university partners had new materials to cover during coaching over time. Also, as a site progressed, coaching needs changed but university partners maintained availability at initial levels.

Data collection was a challenge for sites. Data collection was most successful when collected directly by the university partners such as external coaching data and initial BoQs. Eventually, sites became independent in collecting BoQs, but still required the university partners to analyze the BoQs and create reports for them. Independent collection of coaching logs and eventually EIPPFIs were not successful. Although used internally and for coaching purposes, they were not always fully completed, not entered into the state data system (coaching logs), and not shared with the university partners to summarize and analyze (EIPPFIs). Thus, our findings are limited to what data were available to us.

Having Part C specific PM content was a challenge. In 2015 and 2016, the initial trainings provided by a prior PM partner were the general PM content, geared towards preschool classroom implementation, as Part C specific content was not yet available. The BoQ was available, but little else had yet been formally released. As the current university partners were extensively experienced in the Part C system, they independently adapted PM content for the Part C context and worked closed with sites to apply adaptations. Over time, NCPMI created more content including training and webinar content [34]. Thus, we were supporting PM Part C implementation as the national PM leaders were further developing the model, which naturally led to changes in content over time. However, the university partners worked closely with national leaders and were able to ensure high quality PM support at these sites despite these challenges.

An additional limitation was changing measures over time. As noted above, NCMPI had not yet adapted many of the tools for Part C. A primary PM tool, other than the BoQ, is a standardized classroom observation. However, as Part C takes place primarily during home visits, those tools do not apply. The Part C Early Intervention Pyramid Practices Fidelity Instrument [55] was created in 2019 and gradually introduced nationally, with supporting materials on application process distributed over time. Thus, it took time for university partners to become familiar enough to oversee implementation at sites. Furthermore, as it was far into implementation at these sites, the acceptance of and pivot to a new tool took time. Thus, sites were slow to formally utilize it, and although they did begin to use it with cases as an internal check, we did not reach a level of implementation where data were aggregated and reviewed over time.

Also noted above were different BoQ applications over time. The PM Part C BoQ was updated in 2018, leading to a change in BoQ versions. Additionally, one year one site did not fully complete the BoQ, and in 2022 all sites did not collect the BoQ. In this state, the 2022 school year represented the re-engagement of in person educational programming after almost a full year of virtual programming due the COVID-19 pandemic. Re-entry to in person education was a stressful and hectic time for school districts. Thus, data collection for this project was not prioritized. Despite differences in BoQs and disruptions in data collection, comparison across time remains valid as the approach for the BoQ is to determine the percentage of item in place, partially in place, or not in place. Regardless of the number of items scored the percentage is comparable. Also, the difference between the old and new versions of the BoQ was addition of items but did not reflect significant changes. For the site that only completed 13 items one year, it is difficult to know if the percentage not in place is accurate or if those not completed were predominantly not in place. However, as it was their first year utilizing the BoQ, this more likely reflects learning the tool. All other years, the BoQ data collected was complete by this site.

An additional limitation is a lack of a comparison group. Thus, there are no sites to compare our results to. However, this was a state funded implementation project and was not designed to have control groups. The intention was to use an implementation science approach to evaluate how the PM was applied and if implementation was successful across various sites. In fact, we are able to identify several implementation factors that led to success or challenges with implementation including organizational structure and implementation drivers [58]. Furthermore, consistent with implementation science practices we tracked implementation phases and used cycles of data review to monitor progress and set action plans [40,56]. Thus, as an implementation project, within a complex system, these processes and outcomes are a valid reflection of the successes and challenges of PM implementation within Part C programs.

There are several lessons learned about PM implementation within Part C programs. One is to do a thorough assessment of competing demands and understand how PM implementation fits with other programmatic activities. As noted, all programs were asked to do this in conjunction with implementing additional EBPs and still attend to other requirements. Additionally, having more comprehensive Part C specific materials from the beginning will ensure more consistency in application and data collection over time. Also, having a more systematic approach to readiness for implementation would allow for more systematic selection of candidates and identify where sites need more supports before engaging in implementation [40]. Future applications of the PM within Part C should take these factors into consideration. Currently, the university partners are engaging with the same state for the next SSIP round which again includes implementation of PM, along with Routines Based Interview and Reflective Coaching. However, there are several differences in approach for this round. Sites were asked to apply to participate, which increased the likelihood of program buy-in. Furthermore, the application process included queries for readiness to ensure programs understood the commitment and had resources in place to engage in the work. Additionally, programs are not being asked to simultaneously implement all three EBPs, but rather to learn about each of them and choose one to focus on. Thus, lessons learned from this project are already being applied.

## 5. Conclusions

The PM is an effective model to integrate into Part C programs to increase the capacity to identify and address social and emotional needs. It requires deep commitment from local leadership, support from the state including funding, and a partnership with a PM team that can support implementation and evaluation. With these investments, systems will benefit from increased capacity to identify and address social emotional needs, thereby better serving families within the Part C system.

## Figures and Tables

**Table 1:** Site information.

Site	State Region	Size/Population [42]	Race/ethnic Diversity [42]	Median Household Income [42]	% Living in Poverty [42]	Served in ITP in 2023
**A**	North	Small 104,942	- White 83.71% - African American 7.20% - Hispanic/Latino 4.89% - Asian 5.52% - Asian 1.43%	81,817	9.5%	470 [43]
**B**	Central/Western	Mid 268,755	- White 69.27% - African American 10.76% - Hispanic/Latino 11.33%	106,129	5.1%	752 [44]
**C**	Central	Mid 335,411	- White 48.6% - African American 20.0% - Hispanic/Latino 7.7% - Asian 20.0%	129,549	5.3%	1219 [45]
**D**	Central	Large 1,052,521	- White 42.2% - African American 18.9% - Hispanic/Latino 20.1% - Asian 15.7%	117,345	7.9%	15,370 [46]

**Table 2:** PM training activities.

Site	Topic
2017	
**All Sites**	Overview of infant and early childhood mental health
	Understanding the link between PM, RBI, and Reflective Coaching
	Internal coaching and coaching colleagues
**2018**	
**A**	PM in Part C Booster Training
	Trauma-informed PM for Part C providers and considerations for babies born addicted to substances and their families
**B**	PM in Part C Booster Training
	Trauma-informed PM for Part C providers and considerations for caregiver mental health
**C**	PM in Part C (full training)
**2019**	
**A**	Using the ASQ-SE to support PM
	Implementation for Pre-K
**B**	Using the ASQ-SE to support PM
	Implementation for Pre-K
**C**	Using the ASQ-SE to inform the work for Pre-K
	Trauma-informed PM for Birth–5
	Anxiety and the PM for Birth–5
**D**	PM implementation in Part C
**2020**	
**A**	Review of PM for new Part C providers
	Social Emotional Assessment/Evaluation Measure (SEAM)
**B**	Review of PM for new Part C providers
	Supporting the behavior of children who have anxiety
**C**	Trauma-responsive Pyramid Model for Part B providers
**D**	Review of SEFEL Pyramid Model for new Part C providers
	Supporting behavior of children who have anxiety
	Trauma-responsive Pyramid Model training
	How temperament and attachment impacts behavior
	Caregiver mental health considerations
**2021**	
**All Sites**	Review of PM for new Part C providers
	Training in the Early Interventionist Pyramid Practices Fidelity Instrument (EIPPFI)
**2023**	
**All Sites**	PM in Part C overview (offered to newly hired staff)
**A**	Equity in early childhood

**Table 3:** Coaching Practices Ratings Scales 2017, 2018, and 2019.

Items	2017 (*n* = 19)		2018 (*n* = 49)		2019 (*n* = 19)		
	Mean	*SD*	Mean	*SD*	Mean	*SD*	Range (min.–max.)
1. Acknowledged the [learner’s] existing knowledge, skill, and ability as the foundation for improvement.	2.89	0.94	4.03	0.93	4.17	0.60	1–5
2. Interacted with the [learner] in a nonjudgmental and constructive manner during coaching conversations.	3.47	0.77	4.53	0.86	4.65	0.55	1–5
3. Identified with the [learner’s] targeted skills he or she wanted to learn and a timeline for the coaching process.	2.76	0.75	3.50	1.04	4.00	0.86	1–5
4. Developed with the [learner] a plan for action or practice necessary to achieve targeted skill(s) following each coaching conversation.	2.56	1.34	3.67	1.03	4.14	0.74	1–5
5. Observed the [learner] demonstrate knowledge and understanding of the targeted skill(s) or practice(s).	3.00	0.82	3.81	1.27	4.17	0.99	1–5
6. Observed the [learner’s] use of the targeted skill(s) or practice(s).	3.46	0.52	3.54	1.36	3.93	0.92	1–5
7. Created opportunities for the [learner] to observe [you] the coach and or others modeling the targeted skill(s) or practice(s).	2.92	1.31	3.44	1.23	4.08	0.99	1–5
8. Promoted use of multiple opportunities for the [learner] to practice implementation of the targeted skill(s) or practice(s).	2.24	1.09	3.34	0.97	3.79	0.85	1–5
9. Used both planned and spontaneous opportunities to strengthen the [learner’s] knowledge, skills and abilities.	2.59	1.00	3.40	0.97	4.05	0.71	1–5
10. Asked probing questions to examine the [learner’s] knowledge, skills and abilities.	2.88	0.78	3.93	0.98	4.21	0,68	1–5
11. Prompted [learner] to reflect on his or her knowledge and use of the targeted skill(s) and practice(s) compared with research-based practice standards.	2.38	1.15	3.54	1.07	3.96	0.69	1–5
12. Provided feedback about the [learner’s] knowledge and skills following [facilitated self-] reflection on his or her performance.	2.38	0.89	3.76	0.99	3.88	0.82	1–5
13. Provided and/or promoted access to new information and resources after the [learner] reflected on his or her performance.	2.53	1.25	3.28	1.25	3.86	0.77	1–5
14. Engaged the [learner] in reflection on the usefulness, effectiveness, and need for continuation of coaching.	1.63	1.26	3.13	1.07	4.08	0.84	1–5

**Table 4:** PM Coaching Content.

Site	Frequency	Year and Content
2017		
**A**	Monthly	Review of BoQ to set coaching priorities, developing a structure to support incoming and new staff on the PM training modules, and the development of a single form that structures the formulation of coaching questions for colleague-to-colleague coaching sessions.
**B**	Monthly	Review of BoQ to set coaching priorities, focusing first on assessment and screening tools for social emotional needs for children in care, and the development of a single form that structures the formulation of coaching questions for colleague-to-colleague coaching sessions.
**C**	Monthly	Review of BoQ to set coaching priorities, focusing first on understanding family coaching checklist as a fidelity measure.
**D**	Monthly	Review of BoQ to set coaching priorities, focusing first on understanding strategies for addressing top of the pyramid behaviors within visits, discussed integrating the various needs of their 5 site districts, discussed the history of SSIP work, gave clarity on the family coaching checklist, and discussed initiating a survey of topics they wish to focus on.
**2018**		
**A**	Monthly	Review of BoQ set coaching priorities, focused on how to establish the local leadership team and internal coaching.
**B**	Monthly	Review of BoQ set coaching priorities, focused on how to establish the local leadership team and internal coaching.
**C**	Monthly	Focus on general approaches to coaching and integration of PM with RBI and reflective coaching. Coaching done in conjunction with the state’s RBI lead coach.
**D**	Bi-Monthly	Review of BoQ to set coaching priorities, focusing first on application across five regional teams, local leadership teams, and social emotional screening.
**2019**		
**A**	Monthly	Review of BoQs, systems coaching for application within Part C including use of ASQ to support social emotional screenings.
**B**	Monthly	Review of BoQs, systems coaching for application within Part C including use of ASQ to support social emotional screenings, and how to integrate PM within both Part C and Part B 619 for an integrated Birth–5 system.
**C**	N/A	None
**D**	Bi-Monthly	Review of BoQs, bi-monthly systems coaching for application within Part C including use of ASQ to support social emotional screenings.
**2020**		
**A**	Quarterly	Review of BoQs, systems coaching for application within Part C including use of ASQ to support social emotional screenings, and how to integrate PM within both Part C and Part B 619 for an integrated Birth–5 system.
**B**	Monthly	Review of BoQs, systems coaching for application within Part C including use of ASQ to support social emotional screenings, and how to integrate PM within both Part C and Part B 619 for an integrated Birth–5 system.
**C**	N/A	None
**D**	Bi-Monthly	Review of BoQs, bi-monthly systems coaching for application within Part C including use of ASQ to support social emotional screenings. Emphasis on development of a leadership team in one local district.
**2021**		
**A**	Quarterly	Review of BoQs, systems coaching for application within Part C including use of ASQ to support social emotional screenings, and how to integrate PM within both Part C and Part B 619 for an integrated Birth–5 system. Added coaching on how to use and collect data with the EIPPFI.
**B**	Monthly	Review of BoQs, systems coaching for application within Part C including use of ASQ to support social emotional screenings. Added coaching on how to use and collect data with the EIPPFI.
**C**	Upon request	Systems coaching for application of PM within Part C.
**D**	Bi-monthly	Review of BoQs, bi-monthly systems coaching for application within Part C including use of ASQ to support social emotional screenings. Emphasis on development of a leadership team in one local district.
**2023**		
**A**	Quarterly	Review of BoQs, systems coaching for application within Part C including use of ASQ to support social emotional screenings, and how to integrate PM within both Part C and Part B 619 for an integrated Birth–5 system.
**B**	Monthly	Review of BoQs, systems coaching for application within Part C including use of ASQ to support social emotional screenings. Also, how to include social emotional screening to the Part B 619 system.
**C**	N/A	None
**D**	Bi-Monthly	Review of BoQs, bi-monthly systems coaching for application within Part C including use of ASQ to support social emotional screenings. Emphasis on development of a leadership team in one local district.

**Table 5:** Number of BoQ items in place by site each year.

	Site A	Site B	Site C	Site D
2017 *				
**Not in Place**	12 (50%)	5 (38%)	4 (17%)	10 (33%)
**Partially In Place**	7 (29%)	6 (46%)	7 (29%)	11 (37%)
**In Place**	5 (21%)	1 (8%)	13 (54%)	3 (10%)
2019 **				
**Not in Place**	5 (17%)	8 (27%)	6 (20%)	15 (60%)
**Partially In Place**	11 (37%)	15 (50%)	8 (27%)	10 (33%)
**In Place**	14 (47%)	7 (23%)	16 (53%)	2 (7%)
**2020**				
**Not in Place**	4 (13%)	6 (20%)	3 (10%)	6 (20%)
**Partially In Place**	6 (20%)	10 (33%)	11 (37%)	13 (43%)
**In Place**	20 (67%)	14 (47%)	16 (53%)	11 (37%)
2021 ***				
**Not in Place**	2 (7%)	9 (31%)	3 (10%)	2 (7%)
**Partially In Place**	5 (17%)	4 (14%)	9 (30%)	11 (37%)
**In Place**	23 (77%)	16 (55%)	18 (60%)	17 (57%)
**2023**				
**Not in Place**	0 (0%)	8 (27%)	1 (3%)	19 (63%)
**Partially In Place**	4 (13%)	5 (17%)	12 (40%)	5 (17%)
**In Place**	26 (87%)	17 (57%)	17 (57%)	6 (20%)

* 2017 BOQ had only 24 items, and site B only completed 13 of the 24 items in 2017; ** 2019 and later BOQs had 30 items; *** 2021 Site B only reported 29 items.

**Table 6:** PM implementation stage progress by site.

Site	2017	2018	2019	2020	2021	2023
**A**	Initial Implementation	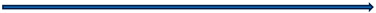				Emerging into Full Implementation
**B**	Initial Implementation	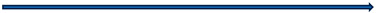				Emerging into Full Implementation
**C**	Installation	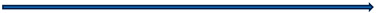				Initial Implementation
**D**	Installation	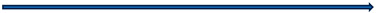				Initial Implementation

## Data Availability

The datasets presented in this article are not readily available because data belongs to the Maryland State Department of Education. Requests to access the datasets should be directed to Margo Candelaria (margo.candelaria@uconn.edu).
